# Spatially variable risk factors for malaria in a geographically heterogeneous landscape, western Kenya: an explorative study

**DOI:** 10.1186/s12936-015-1044-1

**Published:** 2016-01-04

**Authors:** Tobias Homan, Nicolas Maire, Alexandra Hiscox, Aurelio Di Pasquale, Ibrahim Kiche, Kelvin Onoka, Collins Mweresa, Wolfgang R. Mukabana, Amanda Ross, Thomas A. Smith, Willem Takken

**Affiliations:** Laboratory of Entomology, Wageningen University and Research Centre, Wageningen, The Netherlands; Department of Epidemiology and Public Health, Swiss Tropical and Public Health Institute, Basel, Switzerland; University of Basel, Basel, Switzerland; Department of Medical Entomology, International Centre of Insect Physiology and Ecology, Nairobi, Kenya; School of Biological Sciences, University of Nairobi, Nairobi, Kenya

**Keywords:** Malaria, Spatial heterogeneity, Geographically weighted regression, Spatially variable risk factors, Kenya, Socio-economic status, Occupation, Population density

## Abstract

**Background:**

Large reductions in malaria transmission and mortality have been achieved over the last decade, and this has mainly been attributed to the scale-up of long-lasting insecticidal bed nets and indoor residual spraying with insecticides. Despite these gains considerable residual, spatially heterogeneous, transmission remains. To reduce transmission in these foci, researchers need to consider the local demographical, environmental and social context, and design an appropriate set of interventions. Exploring spatially variable risk factors for malaria can give insight into which human and environmental characteristics play important roles in sustaining malaria transmission.

**Methods:**

On Rusinga Island, western Kenya, malaria infection was tested by rapid diagnostic tests during two cross-sectional surveys conducted 3 months apart in 3632 individuals from 790 households. For all households demographic data were collected by means of questionnaires. Environmental variables were derived using Quickbird satellite images. Analyses were performed on 81 project clusters constructed by a traveling salesman algorithm, each containing 50–51 households. A standard linear regression model was fitted containing multiple variables to determine how much of the spatial variation in malaria prevalence could be explained by the demographic and environmental data. Subsequently, a geographically-weighted regression (GWR) was performed assuming non-stationarity of risk factors. Special attention was taken to investigate the effect of residual spatial autocorrelation and local multicollinearity.

**Results:**

Combining the data from both surveys, overall malaria prevalence was 24 %. Scan statistics revealed two clusters which had significantly elevated numbers of malaria cases compared to the background prevalence across the rest of the study area. A multivariable linear model including environmental and household factors revealed that higher socioeconomic status, outdoor occupation and population density were associated with increased malaria risk. The local GWR model improved the model fit considerably and the relationship of malaria with risk factors was found to vary spatially over the island; in different areas of the island socio-economic status, outdoor occupation and population density were found to be positively or negatively associated with malaria prevalence.

**Discussion:**

Identification of risk factors for malaria that vary geographically can provide insight into the local epidemiology of malaria. Examining spatially variable relationships can be a helpful tool in exploring which set of targeted interventions could locally be implemented. Supplementary malaria control may be directed at areas, which are identified as at risk. For instance, areas with many people that work outdoors at night may need more focus in terms of vector control.

Trial registration: Trialregister.nl NTR3496—SolarMal, registered on 20 June 2012

## Background

Across sub-Saharan Africa, malaria remains one of the leading causes of morbidity and mortality with up to 200 million symptomatic cases every year [1]. In Kenya, 75 % of the population is at risk of malaria infection, but due to intensified control efforts the number of malaria cases has decreased two fold in one decade to well under five million annually. Interventions which have contributed to the decline of malaria transmission and mortality are the use of insecticide-treated nets (ITNs), long-lasting insecticidal nets (LLINs), indoor residual spraying (IRS) and treatment of patients with artemisinin-based combination therapy (ACT) [2, 3]. The goal of WHO and Roll Back Malaria (RBM) is to continue the efforts to fight malaria until local elimination and eventually eradication is achieved [4–6].

Since large successes have been realized and many areas have moved into a pre-elimination phase, the epidemiology of malaria is changing [7]. Although malaria transmission has always been geographically heterogeneous, under pressure of current interventions the spatial heterogeneity of malaria becomes more pronounced, typically characterized by areas or clusters of households that persistently have higher proportions of infected individuals compared with the population average. In order to aid the malaria elimination phase, a better understanding of the epidemiology of malaria, considering geographical heterogeneity, is needed [8]. Heterogeneity in malaria transmission is not a new phenomenon [9], but because of improved research methods and the enhanced capacity of information technology, recent studies have more frequently shed light on the smaller-scale geographical heterogeneity of malaria [10–12]. Studies suggest that factors associated with the spatial clustering of malaria include: house structure, human behaviour, environmental, geographical and demographical variables [13–17].

Many studies have investigated clustering and the spatial heterogeneity of malaria risk [18–21] but fewer studies have investigated ways in which relationships of factors influencing this heterogeneity vary over space. Lessons can be learnt from studies that investigated the geographically varying nature of factors on agricultural [22] and environmental [23, 24] outcomes. Relatively few studies have addressed the questions of causes of spatial heterogeneity in health outcomes [25, 26] like malaria [27–30].

In the present study, it is explored whether risk factors for malaria also vary over space. Household and environmental risk factors contributing to malaria prevalence were studied by means of a frequentist non-spatial risk model and clusters of elevated malaria risk were identified through scan statistics. The final aim of this study was to investigate the spatial heterogeneity in relationships between malaria prevalence and associated risk factors by Geographically Weighted Regression (GWR). The added value of using this geostatistical model is explored, and the advantage compared to a standard linear regression model is evaluated.

The study is embedded as part of a baseline study in a large malaria vector control trial (SolarMal) on Rusinga Island, western Kenya [31]. The SolarMal trial aims to reduce malaria transmission on Rusinga Island by mass trapping of malaria vectors with odour-baited traps (OBTs), which contain a blend of organic volatiles that mimic a human odour [32]. Through daily removal trapping the project aims to reduce malaria vector populations and eventually decrease malaria transmission. The analysis of spatial heterogeneity of risk factors for malaria can give a better understanding of malaria epidemiology and can be of value for programme managers who want explore targeting interventions to specific geographical locations.

## Methods

### Study site and population

**Fig. 1:**
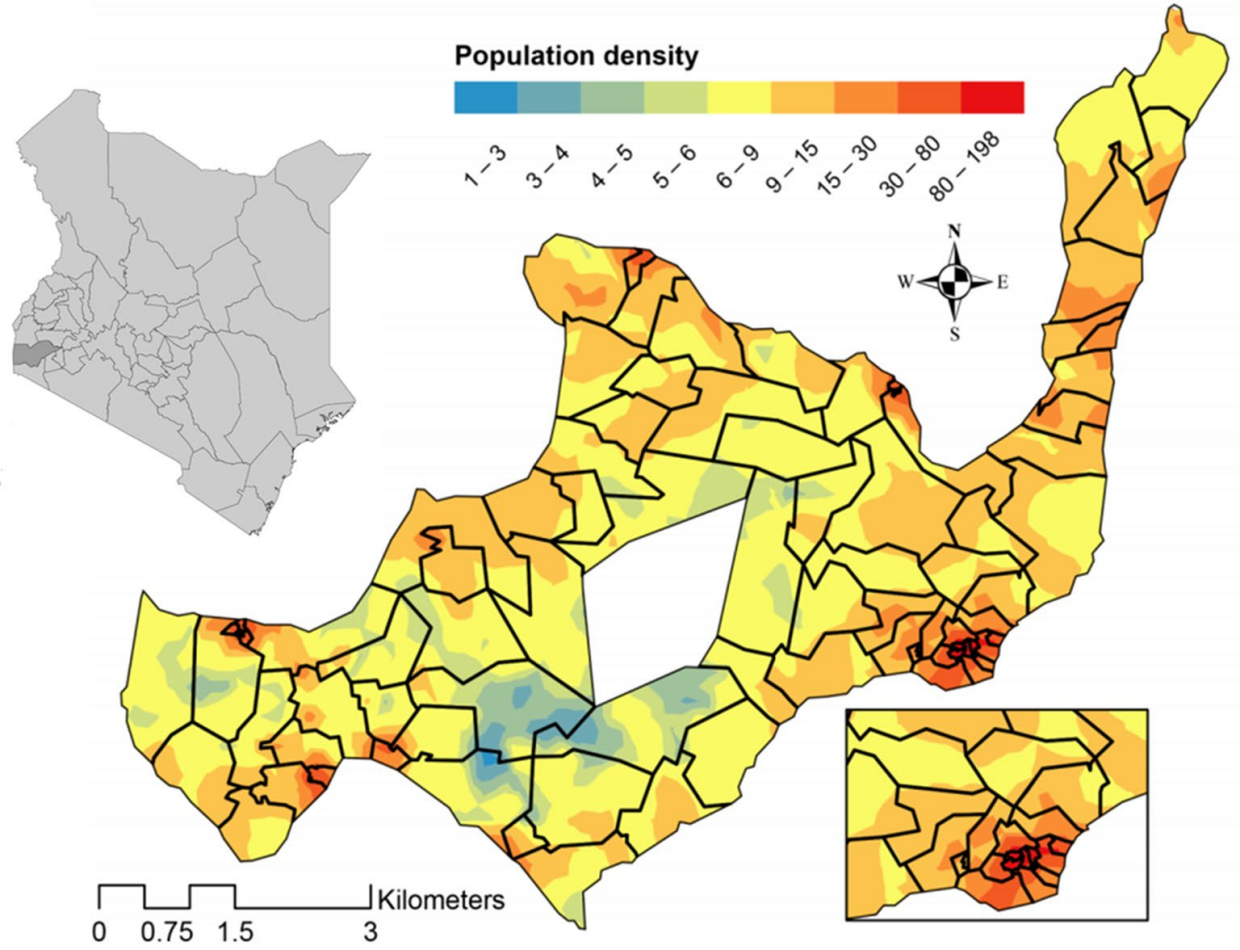
Kenya with the Homa Bay County highlighted where the study site is located. Rusinga Island is mapped showing population density per 250 m^2^ with the boundaries of 81 clusters with equal numbers of households. The *blank space* in the *centre* of the map is an uninhabited hill and the densely populated south-east is magnified—depicted in the *bottom right of the figure*

Rusinga Island is located in Lake Victoria off the shore of western Kenya (between 0°20′51.53″–0°26′33.73″ South, and 34°13′43.19″–34°07′23.78″ East). The island is located in Mbita sub-county, under the administration of Homa Bay County in western Kenya (Fig. 1) and is connected to Mbita Point on the mainland by a causeway. Rusinga Island has a land surface of nearly 44 km^2^ with most of the residential areas situated between 1100 and 1200 m above sea level around the lakeshore of the island. This region experiences a bimodal pattern of rainfall, with the longer rains usually starting in March and ending in June and a shorter rainy season from November to December. Average temperatures range from 20 to 29 °C in the rainy season and from 25 to 34 °C in the dry season.

On Rusinga Island, the population is traditionally part of the Luo tribe. The principal occupation is fishing and labour associated with fishing, otherwise many of the inhabitants are involved in rain-fed subsistence agriculture. Malaria transmission occurs throughout the year, with peaks in transmission late in the rainy seasons when parasite prevalence is approximately 30 % across the population [28]. *Plasmodium falciparum* is the most prevalent species of malaria in western Kenya accounting for 98 % of the cases and the malaria transmitting vectors are *Anopheles funestus* and to a lesser extent *Anopheles gambiae* s.s. and *Anopheles arabiensis* [33–35].

### Field set up

The SolarMal project is based at the Thomas Odhiambo Campus of the International Centre of Insect Physiology and Ecology (TOC-icipe) in the village of Mbita Point, one kilometre from the causeway which connects the island to the mainland. Meteorological data such as daily temperature and precipitation were obtained from the Suba meteorological field station at Rusinga Island (0°24′19.28″ South and 34°08′51.94″ East). A health and demographic surveillance system (HDSS) was set up to visit every individual living on Rusinga Island three times per year. A census enumeration survey, conducted from May to July 2012 recorded 23,337 individuals residing in 6954 residential structures (henceforth termed houses) divided into 4063 economically independent households. During the census HDSS round, the coordinates of all residential structures, as well as public buildings, were recorded. Fieldworkers were equipped with mobile tablet computer devices (Samsung Galaxy Tab 2, 10.1) with in inbuilt global positioning system (GPS) receiver for the data collection. All individuals were asked to provide their full name, sex, date of birth, main occupation and their relationship to the head of household. An individual was considered eligible for participation in the study when he or she intended to live for at least 6 months on the island. Data collection and handling was conducted using general structured questionnaires in the OpenHDS data collection and management platform. Data were transferred on a daily basis to a secured local server enabling researchers to work with a completely digital near real time database. Clean data were deposited in a MySQL database. During baseline studies one HDSS update survey was conducted from January to June 2013. For the rollout of the intervention the island was divided into 81 geographically contiguous clusters with 50–51 households per cluster. The households were allocated to clusters according to a travelling salesman algorithm by which the shortest imaginary route connecting every household on the island was identified. A new cluster was created after every 50–51 households [36] (Fig. 1). 81 clusters is a sufficient number of units to carry out regression while a sample from approximately 50 households provides enough statistical power to estimate the true value for a cluster.

### Malaria surveillance

During the baseline period before the rollout of the intervention commenced, two parasitological prevalence surveys were conducted in a cross section of the study population. Households were randomly selected for inclusion in each prevalence survey to the point where 10 % of the population was included. All members of selected households were informed in advance of the date and time of the survey and were invited to assemble at a public place such as a church or a school near their home for malaria testing. In total, residents of 790 randomly selected households were sampled, covering 1223 houses. The first survey examined 1822 individuals (7.8 % of the total island population) and was carried out during the start of the short rainy season starting from September and finishing in November 2012. A second prevalence survey examined 1810 individuals (7.7 % of the total population) and was conducted from February to April 2013. Individual body temperature was measured by means of a Braun™ IRT 3020 ear thermometer. A drop of blood was obtained through a finger prick and directly tested for antigens of malaria parasites using an SD BIOLINE™ Malaria Ag P.f/Pan (HRP-II/pLDH) Rapid Diagnostic Test (RDT). The SD Bioline RDT kit results distinguish between infection with *P. falciparum* and other *Plasmodium* species. However, tests results with more than one positive reading or indicating multiple species of *Plasmodium* were pooled. If the individual tested positive for malaria antigens, an appropriate dose of Coartem^®^ (Artemether/lumefantrine) was provided free of charge.

### Household information

Besides the demographic information, Table 1 lists variables recorded concerning the house structure and existing malaria prevention behaviour and whether they were derived from the level of the individual or the household. An index of socioeconomic status (SES) was constructed by means of a principal component analysis producing tertiles of socioeconomic status on basis of six variables, [37] as used in the Kenyan national malaria indicator survey. [38] The variables used were: whether the dwelling was owned or rented, whether agricultural land was owned, highest education level of the head of household, location of the kitchen, the wall structure and the floor cover. Every individual was categorized into one of the three SES classes: high, intermediate and low. Data were transformed into continuous variables with means calculated per cluster based Means of variables per cluster were constructed either on basis of individual level data or household level data (Table 1). Sex was expressed as the proportion of males per cluster; age was divided into three dummy variables, the proportion of children under 5 years old, between 5 and 15 years and above 15 years; occupation was categorized as the proportion of people in a cluster having an outdoor occupation; house ownership is the proportion of houses that are owner-occupied rather than rented; for SES the two lowest categories were pooled so a dummy variable remained for high SES and not a high SES, the percentage of people having the highest and the lowest socio-economic status; eaves as the percentage of houses with open eaves; and condition of nets is the proportion of people sleeping under an intact net.

**Table 1:** Variables considered for the global regression model of malaria prevalence

Variable	Description for GWR per project cluster
Sex	% males
Age1	% of children under 5 years old
Age2	% of children between 5 and 15 years old
Age3	% of people above the age of 15
Occupation	% outdoor occupation
People per sleeping room	Mean people per sleeping room
People per house	Mean people per house
Screened eaves	% houses with open eaves
Condition of bed nets	% bed nets without damages
House sprayed last 12 months	% sprayed houses in last 12 months
Nets per person	Mean number of nets per person
Socio economic status1	% of people with highest SES
Socio economic status2	% of people with lowest SES
House ownership	% of houses owned
Population density	Mean population density
Mosquito exposure	Mean malaria mosquito catches per house
NDVI	Mean NDVI
TWI	Mean TWI
Distance to lake	Mean distance to the lake
Elevation from lake	Mean elevation from lake
Distance to clinic	Mean distance to nearest health clinic

*SES* socio economic status, *NDVI* normalized difference vegetation index, *TWI* topographic wetness index

### Entomological monitoring

Monitoring of mosquitoes took place across five consecutive rounds from September 2012 until June 2013, selecting 80 households per round, each time by means of a simple random sample, with replacement, of all households on the island. Mosquitoes were collected inside and outside selected households using odour-baited MM-X traps (American Biophysics Corporation, RI, USA) [32, 39]. Data from the first, second, fourth and fifth rounds of surveillance (September to November 2012 and March to June 2013) were pooled as they corresponded temporally with the two baseline malaria prevalence surveys. In total entomological data from 353 households was included in this study. The total number of female anophelines caught inside and outside each household was pooled as a single observation for that particular household.

### Geographical variables

A multispectral QuickBird image, taken on 17/03/2010 with a spatial resolution of 2.4 m, was obtained through DigitalGlobe^®^. Initially, the image was used for geo-referencing of residential and public structures and infrastructure. The image was geo-referenced, radio-metrically corrected, corrected for sensor and platform-induced distortions, and was ready for orthorectification. Orthorectification was performed using a Digital Elevation Model (DEM). The DEM used was an ASTER GDEM 2, the geographical coordinate system was referenced to the 1984 World Geodetic System (WGS84). Several geographic variables were derived for each household using the image and DEM: elevation relative to lake, distance to lake, distance to nearest clinic, population density, the Normalized Difference Vegetation Index (NDVI) and the Topographic Wetness Index (TWI). The NDVI is a commonly used indication of greenness and is calculated based on the values of the red and near infrared spectral bands within a radius of 250 m. The TWI defines the wetness of an area and combines the upstream area with the local slope expressed as the number of cells ‘upstream’ of cells measuring 30 × 30 m (900 m^2^). Population density measures were calculated within a radius of 250 metres. All the geographical variables per household were averaged per project cluster for data analysis and the analysis was at cluster-level. Geographic data and variables were pre-processed, compiled and displayed using ArcGIS (ArcGIS 10.2.1, ESRI Inc., Redlands, CA, USA).

### Statistical analysis

For this analysis the measurements of both prevalence surveys were pooled and the mean malaria prevalence per project cluster on basis of individual RDT outcomes was analysed and mapped with smoothing using areal interpolation technique. Areal interpolation is a kriging-based interpolation method that considers involvement of polygons of different shapes [40]. A Gaussian distribution for data averaged over polygons was used to produce semivariograms. Semivariograms were then used to investigate the degree of spatial variation; the model function was chosen which captured the most empirical data points within its confidence intervals.

Unlike the regression analyses that are based on continuous household or individual data of project clusters (Table 1), the detection of potential ‘hot spots’ of malaria cases were analysed with a binomial distribution on an individual level, with the outcome variable malaria positive or negative. Kuldorff spatial scan statistic analyses were performed (SaTScan, v9.1.1) [41, 42] using a circular window that gradually scans the map of the island, quantifying the number of observed and expected observations within the window for every house. Within each circle, values in a radius around each household were compared to the expected values and a likelihood ratio test was subsequently performed. P values were obtained by 999 Monte Carlo replications and when p values were ≤0.05, houses in this circle were considered to be part of a significant hot spot of elevated malaria prevalence. The maximum scan window was set at 1.5 km and a maximum of 50 % of the population was allowed in one possible hot spot.

Stationary epidemiological risk models assume that observations are geographically independent. These ‘global’ models assume that malaria and the coefficients of predictor variables apply to the whole island [43]. Outcomes can be biased because the models do not account for spatial dependence considering that the relationship of risk factors for malaria can vary over space, such as demographical and environmental features [44]. In order to gain an enhanced insight into variation in malaria outcomes, incorporating potential spatial dependence of predictor and dependent variables is vital where disease patterns are spatially heterogeneous. Moreover, to effectively capture spatially variable associations between risk factors and malaria outcomes, regression coefficients may vary locally as well. To include these considerations of spatial non-stationarity a geographically weighted regression (GWR) model was deployed [45]. A log transformation was performed to normalize the slightly positively skewed malaria prevalence data on cluster level.

To explore which predictor variables to include in the GWR model, a global multivariable regression (stationary model) was initially performed. In adopting the best model for explaining log transformed risk several other model features other than the best goodness-of-fit or statistical significance of predictors were looked at. Next, the assumption of normally distributed residuals of the estimated outcome (tested by the Jarque–Bera test) was tested as the model prediction function relies on normally distributed unexplained variance. The predictor variables that were included cannot have any multicollinearity in order to prevent duplication of capturing any predictive effect (indicated by a Variance Inflation Factor of <7.5). Moreover, regression residuals need to be randomly distributed to make sure that observed relationships are not inflated because the observed minus the predicted values are not independent from each other [46]. Regression residuals were examined for residual spatial autocorrelation (RSA). Furthermore, a test to detect heteroscedasticity was carried out to get an idea of heterogeneity in the relationship between the predictor and dependent variables (Breusch–Pagan statistic). The model that satisfied all these requirements and had the highest R^2^ was selected for further analysis in a GWR model. The model did not control for possible correlated observations.

In relationships between dependent and independent variables the GWR produces local linear regression models. The coefficients in a standard linear regression model are assumed to be the same at every location, whereas regression coefficients of a GWR model are attached to each individual location, in this case the location of a central point of a cluster [47]. Coordinates of project clusters were determined by taking the centroids of the polygon features. The GWR regression model is thus:

1 $$y_{i} = \beta_{0} + \mathop \sum \limits_{k = 1}^{p - 1} \beta_{k} x_{ki} + \varepsilon_{i}$$

where every observation *i* has its own set of coordinates, $y_{i}$ is the cluster prevalence and $x_{ki}$ is the value for a covariate *k* for observation *i*, $\beta_{0}$ is the intercept, $\beta_{k}$ is the coefficient estimate for a covariate *k*, and $\varepsilon_{i}$ is the random error for observation *i*, and *p* is the number of regression coefficients to be estimated. Estimations of predictor variables were obtained using subsets of data in a radius around observed geographical data points. Weights were applied to the subsets of observations, with a Gaussian decaying influence as distance increases. The radius determining the distance at which neighbouring data points influence the local models is known as the kernel bandwidth. For this analysis an adaptive kernel function (bi-square) was chosen instead of using a fixed radius; it considers a number of neighbouring data points leading to weights:

2 $$W_{ij} = \left\{ {\begin{array}{*{20}c} {\left[ {1 - \left( {\frac{{d_{ij} }}{{d_{iN} }}} \right)^{2} } \right]^{2} } \\ 0 \\ \end{array} } \right.$$

where $W_{ij}$ is the weight of data at location *j* estimated for location *i,*$d_{ij}$ is the distance between locations *i* and *j,*$d_{iN}$ is the distance to the spatial neighbours of location *i* and *N* is the number of neighbours considered ${\text{W}}_{\text{ij}}$ takes zero for locations that are farther away from location i than the kernel bandwidth set. The optimal bandwidth and the associated weighting function were obtained by choosing the lowest score of the corrected Akaike information criterion (AIC*c*). It seeks parsimony, finding a balance between model fit and amount of parameters in the model. The AIC*c* was obtained by reducing the estimation error of our dependent outcome to a minimum and is:

3 $${\text{AIC}}c = 2n\log e \left( {\hat{\sigma }} \right) + n\log e(2\pi ) + \left\{ {\frac{n + tr(S)}{n - 2 - tr(S)}} \right\}$$

where $\hat{\sigma }$ is the estimated standard deviation of the error, and *tr*(*S*) is the trace of the matrix of covariates.

A set of local goodness-of-fit statistics was derived by plotting the local R^2^ per cluster. Furthermore, local coefficients and p-values belonging to predictor variables yielded were plotted to explore the geographically varying relationships with malaria prevalence. A semivariogram of regression residuals is constructed to explore the spatial structure of the model. To examine the final GWR model for possible spatial autocorrelation in the residuals (RSA), a Moran’s I test was performed on the residuals between observed and predicted values of malaria prevalence. Finally the model predictions were validated by means of exhaustive cross validation. Many different samples of training and a validation sets were considered to validate predictions in every cluster.

Special attention is given to the issue of local multicollinearity because GWR outcomes can be heavily biased, and local coefficients can become inflated if different predictor variables have similar geographical patterns [48]. Local multicollinearity is assessed by the condition number. This number increases if predictor variables show similar patterns, and when this number is above 30, the model is assumed to be unstable and unreliable.

Statistical analysis and model building were performed using R software (RStudio, Inc.© version 0.98.1102 package *spgwr*), GWR4© (Newcastle University, UK) and ArcGIS (10.2.1, ESRI Inc., Redlands, USA).

### Ethical clearance

Ethical approval was obtained from the Kenyan Medical Research Institute (KEMRI); non-SSC Protocol No. 350. All participants were provided with written and oral information regarding the project aims, the ongoing demographic and entomological surveillance activities, the implementation of the intervention, and the collection and use of blood samples. Adults, mature minors and caregivers of children provided written informed consent in the local language agreeing to participation in the SolarMal project activities.

## Results

**Fig. 2:**
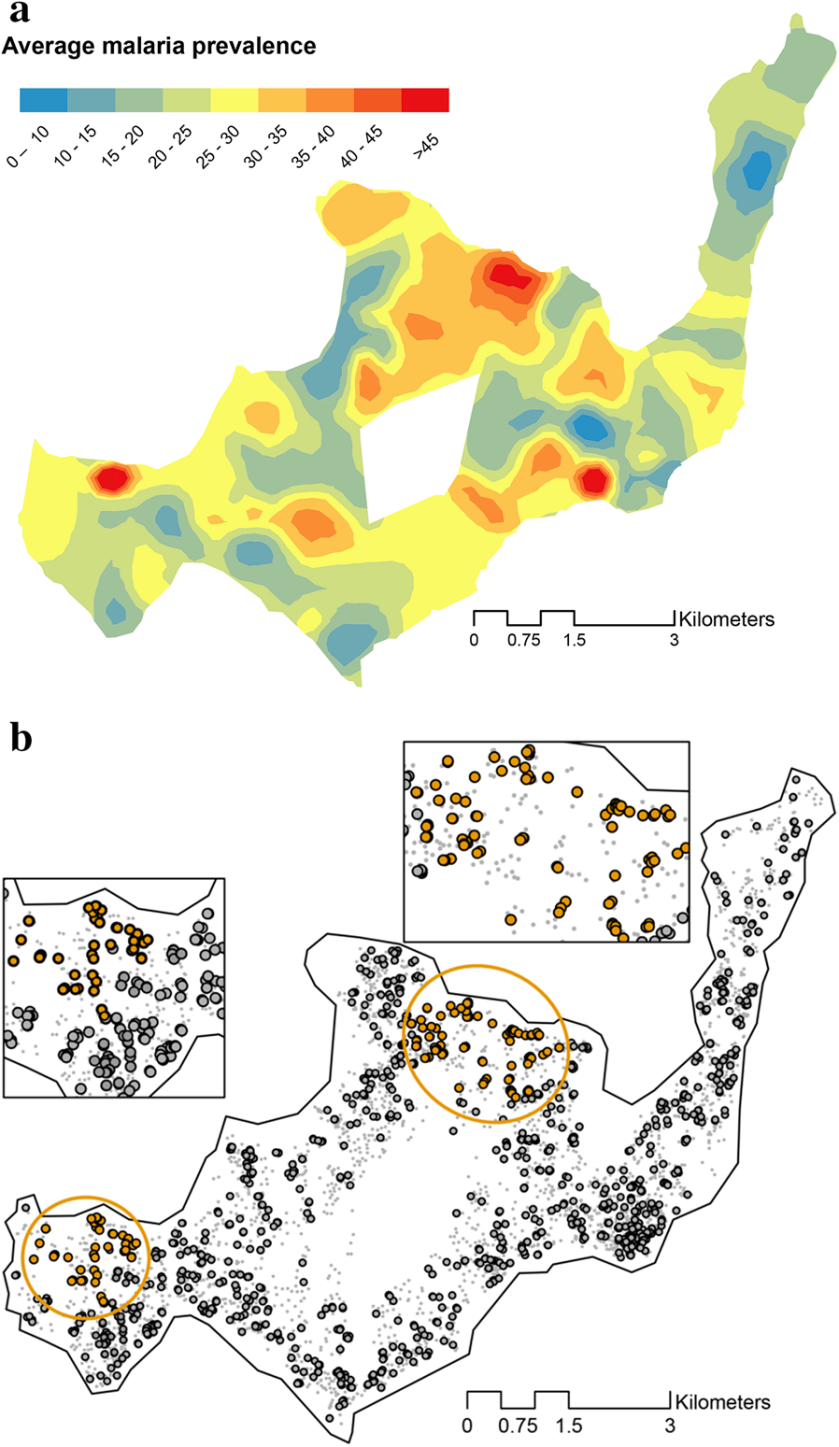
**a** Mean malaria prevalence per cluster on the basis of sampled individuals across Rusinga Island using Aerial interpolation. **b** Map of Rusinga Island showing two clusters of households (*orange dots*) with significantly elevated levels of malaria prevalence. The primary cluster is located at the central north of the island; a secondary cluster is covering an area to the west. Figure 2 **a** would suggest another cluster of malaria in the south-east, however prevalence in this area is not significantly greater than in neighbouring areas. The *grey dots* **b** with black outlines are the sampled houses in the prevalence surveys; the paler *grey dots* indicate all houses on the island

Possible hot spots of elevated malaria risk were identified by plotting the malaria prevalence per project cluster and smoothed with the areal interpolation technique (Fig. 2a). The island-wide malaria prevalence was 24 % and the prevalence per cluster varied between 9 and 75 %. Subsequently a SatScan analysis was conducted revealing two significant hot spots of malaria; one in the west and one in the central north of the island (see Fig. 2b). The primary hot spot of malaria is located in the central north of the island, the observed number of cases here was significantly higher than predicted from island-wide values (Table 2). The risk of malaria in this hot spot is almost three times higher than for areas outside this hot spot (RR = 2.65, LLratio = 42.509, p value ≤0.0001). Furthermore, a secondary hot spot of malaria was identified in the west of the island with more than twice the risk for malaria infection (RR = 2.12, LLratio = 20.399, p value = 0.001).

**Table 2:** Summary results of hot spots detected by SatScan

Cluster	Relative Risk	LL ratio	P value	Number of individuals	Expected infected individuals	Infected individuals
1	2.65	42.51	<0.0001	298	29	69
2	2.12	20.40	0.001	212	23	46

### Global linear regression model

The multivariable global linear regression (GLR) model explains 26.8 % (R^2^) of the total variation between project clusters in malaria prevalence. The model and statistics on model assumptions are summarized in Table 3. The null-hypothesis of no residual spatial autocorrelation (RSA) in the model is maintained with the Moran’s I statistic not being significant, showing that the regression residuals are randomly distributed and not missing key explanatory variable. The Breusch–Pagan statistic examines whether the relationship of predictor variables with malaria prevalence is similar around the island; heteroscedasticity is clearly present (with a p value of 0.03). Furthermore, the residuals of the outcome variable are approximately normally distributed indicating no deviation from the distributional assumptions of the model.

**Table 3:** Summary results for best non-spatial linear regression model for malaria prevalence

Variable	Coefficient	Std error	P value	Robust Std error	Robust P value	VIF
Intercept	−0.827	0.059	<0.0001	0.061	<0.0001	–
Outdoor occupation	0.566	0.195	0.005	0.200	0.006	1.16
Highest SES	0.240	0.098	0.017	0.101	0.020	1.55
Population density	−0.004	0.001	<0.0001	0.001	0.001	1.38

Statistic	Value					
Joint Wald Statistic	18.75; p = 0.001					
Moran’s I	0.45; p = 0.21					
Breusch–Pagan statistic	8.86; p = 0.03					
Jarque–Bera statistic	4.05; p = 0.13					

*SES* socio economic status, *VIF* variance inflation factor

Because heteroscedasticity is significantly present in the GLR model, the robust p value and standard errors were used to assess the relationships of the predictor variables with malaria prevalence. Outdoor occupation is the strongest significant predictor in the model with a coefficient of 0.57 (and a p value of <0.0001). Furthermore, belonging to a household with a high SES is positively associated with malaria prevalence with a significant coefficient of 0.24 (and a p value of 0.02). A third significant predictor variable is population density, although the coefficient was only −0.004 (p value of 0.001). All predictor variables in the final global model were tested for multicollinearity, and all are well below the threshold of 7.5 (Table 3).

### Geographically weighted regression model

The predictor variables of the GLR model (outdoor occupation, SES and population density) were incorporated into a geographically weighted regression model. To determine the number of neighbouring clusters for local regression the bandwidth with the lowest AICc was chosen. The bi-square adaptive kernel function looks at an adaptive number neighbours and the influence of these neighbours decays following a Gaussian distribution so that closer observations have most weight. So local regression for clusters that have few data points adjacent, will include clusters farther away. Comparing the global and the local model shows that the GWR model performs better than the GLR model with an AIC (a measure to compare model quality) value of −43.8 versus −40.2 (Table 4). Moreover, the GWR model fits considerably better taking into account non-stationarity. The capability of the GWR model to predict malaria prevalence on basis of the selected predictors is best expressed by looking at the R^2^, improving the model fit from 27 to 69 %. Other indications that show a better fitting and predicting model are the residual sum of squares and the −2 Log Likelihood, both statistics are less than half compared to the local model.

**Table 4:** Comparison between global regression and GWR model

Variable	GLR	GWR
AIC	−40.86	−43.18
Moran’s I	0.45; p = 0.21	0.23; p = 0.25
R^2^	0.268	0.694
Residual sum of squares	2.53	0.985
−2 Log likelihood	−50.86	−127.26

Model fit is compared with AIC, explanatory power of the models is compared by R^2^ and the Moran’s I of residuals indicates the degree of spatial autocorrelation

**Fig. 3:**
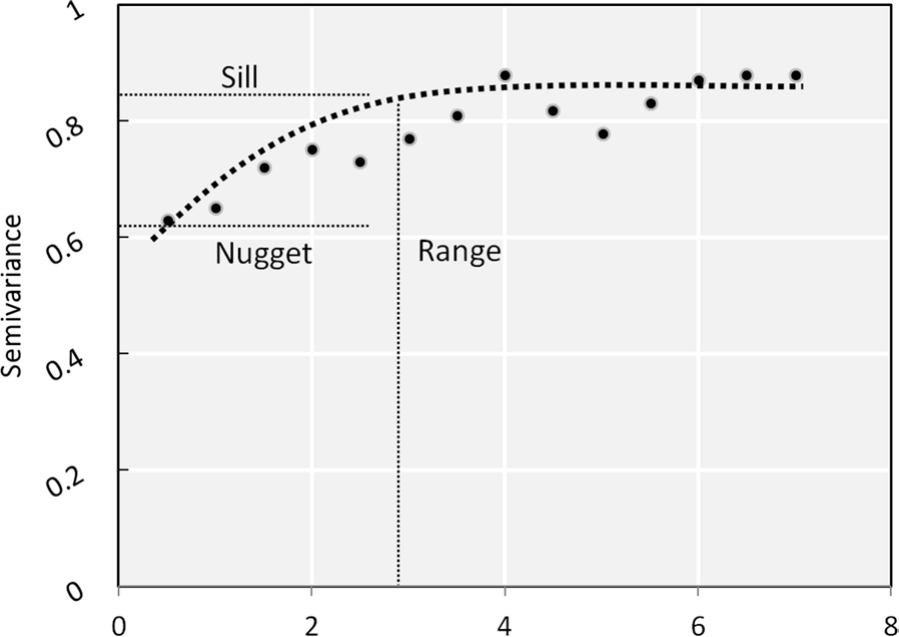
Semivariogram of the residuals of the final GWR model, with the *dotted line* showing the fitted value. The semivariance is shown on the y-axis. The semivariance of the residuals between households starts at 0.61 (nugget) demonstrating some spatial autocorrelation on distances up to 2.7 km (range). Beyond this threshold the semivariance is high and stabilizes at 0.825 (sill) indicating minimal RSA

**Fig. 4:**
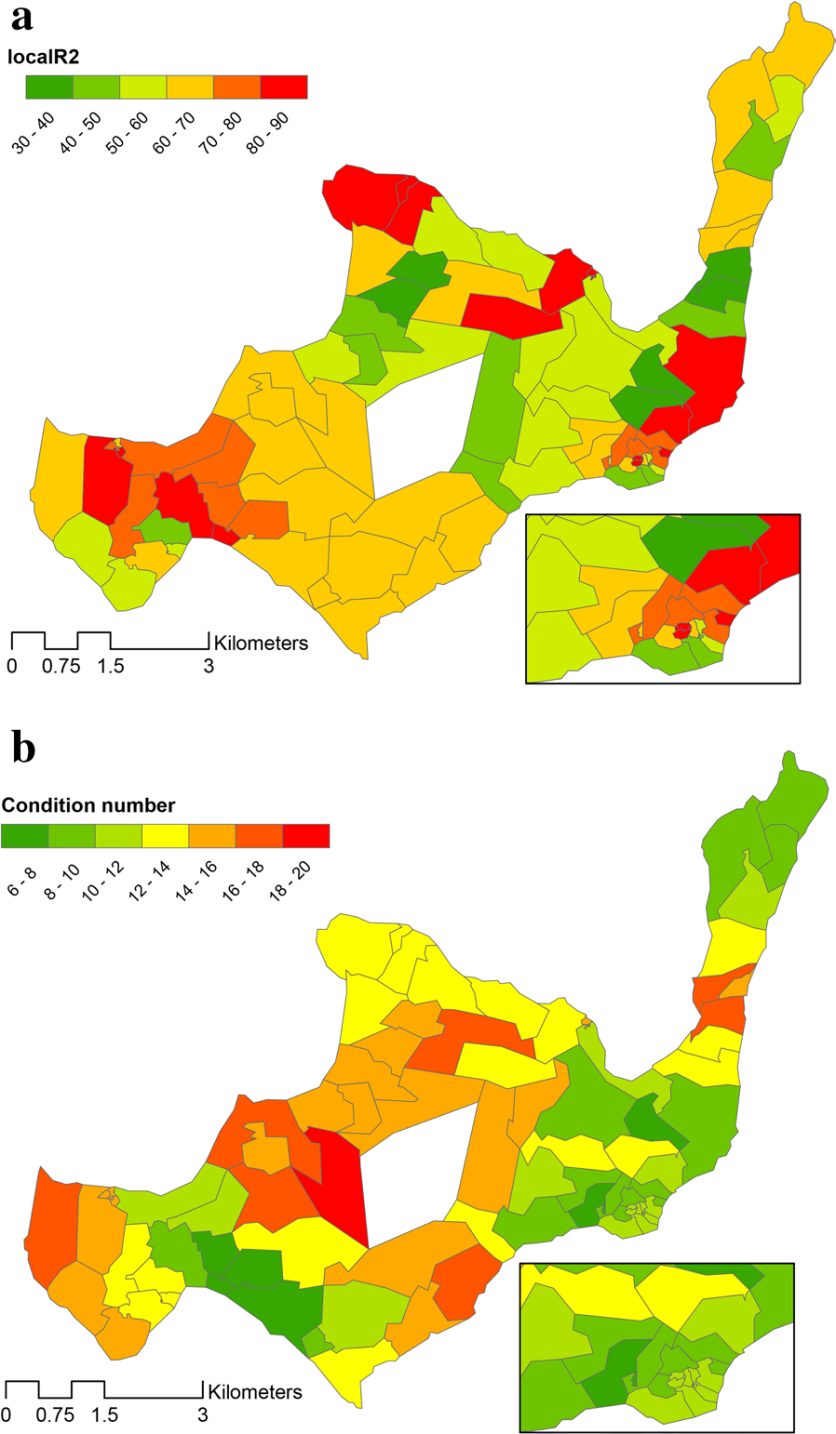
**a** Goodness-of-fit statistics indicate how well the GWR model fits per cluster, expressed by R^2^ and **b** Multicollinearity per cluster, expressed by the condition number. A higher condition number indicates an increased degree of multicollinearity

Exploring the spatial structure of the model residuals with an anisotropic averaged semivariogram shows that the distance up to which RSA occurs (the range) is 2.7 km (Fig. 3). The sill has a value of 0.825, indicating that the variance of residuals between households beyond the value of the RSA range is fairly high. Within the range the variance starts from 0.61 (the nugget), demonstrating that the degree of RSA is not pronounced. Spatial autocorrelation in the residuals of the final GWR model was then assessed by a Moran’s I test and this actually directed to some RSA. Nevertheless this yielded a p-value of 0.25, thus the null hypothesis of no significant RSA was maintained. R^2^ values per cluster vary between 32 and 87 % with a mean of 63 % (Fig. 4a). Local multicollinearity assessed by the condition number yields values of between 6.7 and 19.2 with a mean of 12.9, indicating that the model is marginally affected by multicollinearity (Fig. 4b). Cross validation of the predicted malaria values with the measured values yielded predictions for 74 of 81 project clusters that were statistically significant.

**Fig. 5:**
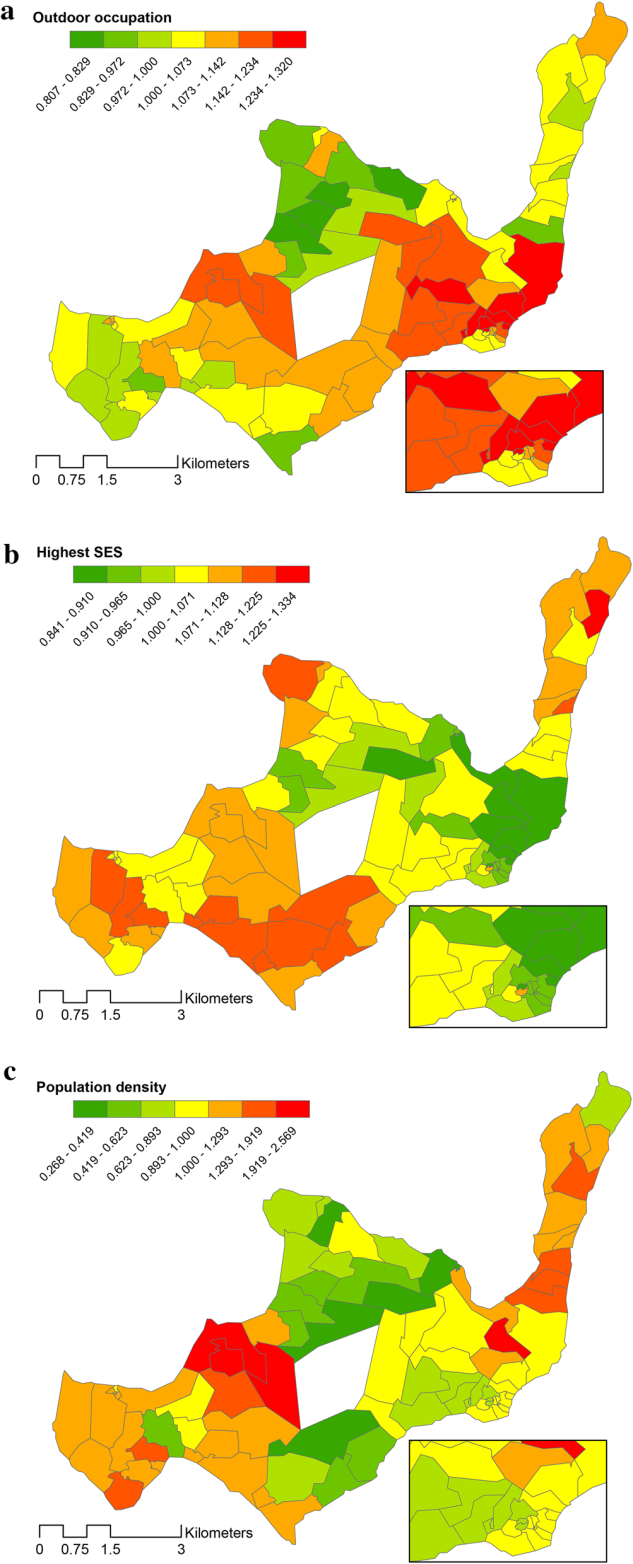
Geographically varying coefficients expressed as the relative risk per cluster for predictor variables of malaria prevalence in the final GWR model. **a** Outdoor occupation, **b** highest SES, **c** population density

Geographically varying effects of outdoor occupation, SES and population density in the GWR model are illustrated in Fig. 5. Regression coefficients were back-transformed after the initial log transformation of malaria prevalence in the model, and presented as exponentiated coefficients. This is interpreted for the highest SES category as the relative malaria risk compared to being in a lower SES category or having another occupation. The same interpretation applies to the outdoor occupation variable. For average population density the interpretation of the coefficient is best expressed as the increase in malaria risk for every one person increase in the average number of individuals per 250 m^2^.

**Fig. 6:**
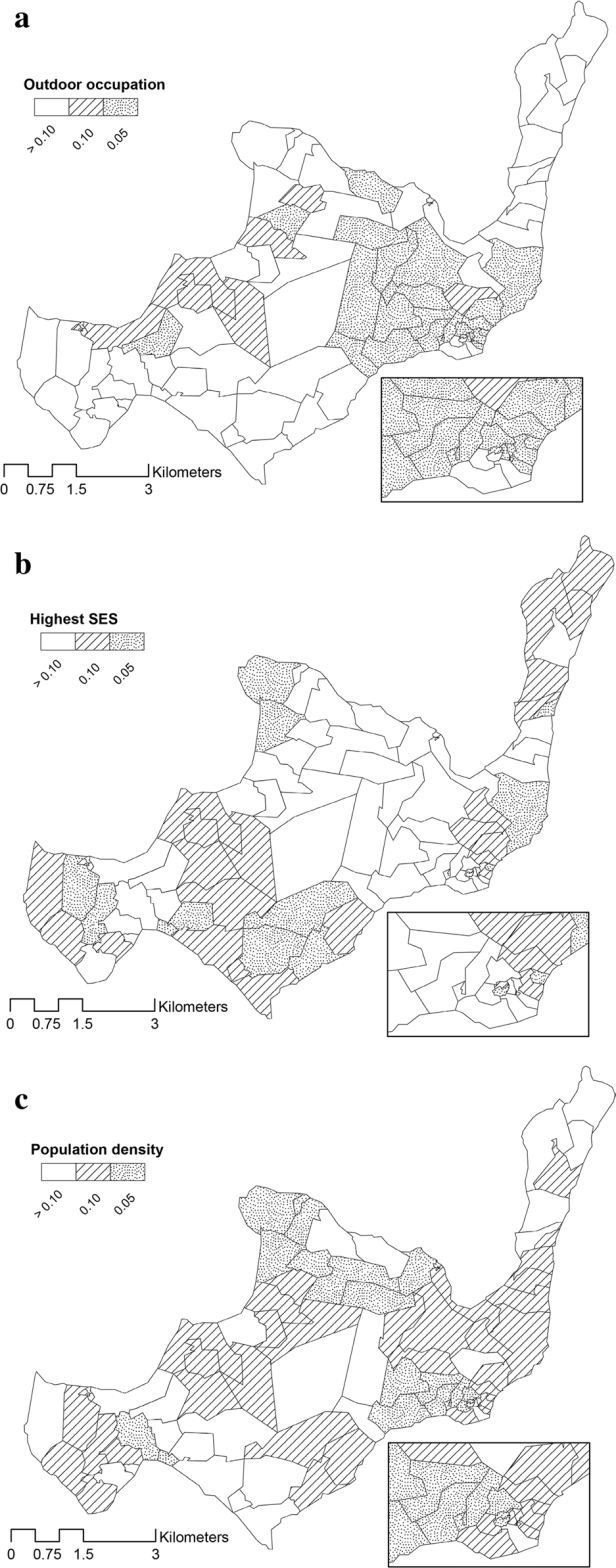
Geographically varying values of significance per cluster for predictor variables of malaria prevalence in the final GWR model. **a** Outdoor occupation, **b** highest SES, **c** population density

Population density, outdoor occupation and highest SES differ in having a positive or negative association with malaria prevalence. Coefficients of each variable can have a positive or negative association and the direction of the association varies depending on the local values of those explanatory variables. Coefficients that are equal to one indicate a similar malaria risk compared to the surrounding comparator clusters, whereas coefficients risks above one demonstrate an increased risk for malaria. The coefficients of malaria for population density varied between 0.268 and 2.569 indicating that the association between malaria and population density could be positive or negative depending on the area of the island. The variation in coefficients of malaria for those in the highest SES group ranged between 0.841 and 1.334, also indicative of a negative association in some areas of the island but a positive association in other areas. Outdoor occupation also had a spatially variable association with malaria, with exponentiated coefficients ranging between 0.807 and 1.320. P values of regression coefficients of all three explanatory variables also vary over space (Fig. 6), indicating that the statistically significant relationships were not equally strong everywhere on the island.

## Discussion

Over the past decade large reductions in malaria have been achieved, yet the current distribution of malaria is still spatially heterogeneous [7, 49]. Considerable research is currently being conducted to find tools for malaria control that are able to target residual malaria transmission, in order to reach the goals set by the RBM initiative to eliminate malaria where possible, or reduce it to a minimum [50, 51]. Established interventions such as LLINs, IRS and case management have proven to be effective but this one size fits all strategy is not appropriate when moving into the elimination phase [8]. These existing methods will need to be complemented by novel tools, which may entail interventions targeting local geography, demography and societal context [6]. Exploring locally varying relationships of risk factors for malaria may aid in exploring and eventually targeting appropriate interventions. Traditional descriptions and models report on the progressively heterogeneous nature of malaria transmission, but analyses reporting on risk factors for malaria and disease usually ignore spatial heterogeneity of the underlying risk factors of disease [52].

In exploring spatially varying relationships of risk factors for malaria, factors that are directly related to malaria risk as well as proxy factors were used. Socioeconomic status, screened eaves and condition of bed nets are examples of factors directly influencing malaria risk, whereas distance to nearest clinic and environmental variables as TWI and NDVI can have an indirect effect because of access to anti-malarials or proximity to possible breeding sites for malaria vectors. The GLR model explained 27 % of the spatial variance in malaria prevalence, however GWR analysis greatly improved model fit to 69 %. A better fit by the GWR model is confirmed by a reduction in the residual sum of squares as well as an increased likelihood when comparing the global and the local model (Table 4). Local estimations of model fit did vary somewhat over the island (Fig. 4a), and whilst there are several areas where the model does not fit more than 50 %, in all study clusters an improved fit using the GWR was observed compared with the global model.

Outdoor occupation and activity at night have previously been associated with higher risk for malaria [53, 54]. In the case of Rusinga Island, many people are involved in fishing and labour related to fishing, and these activities are generally performed in shifts during the night. It is known that in between shifts, fishermen spend their time around fishing beaches close to their home with little or no protection against biting malaria mosquitoes. It is during the night that *Anopheles gambiae* s.l. and *An. funestus* mosquitoes exhibit their peak host-seeking behaviour, biting mostly indoors but also outdoors [55], thus people who are active at night are expected to be at increased risk for receiving infective mosquito bites. Spatial heterogeneity of outdoor occupation in the south-east of the island is characterized by a large area where having an outdoor occupation leads to increased risk of malaria. This is the area of Rusinga with the highest proportion of fishermen. Malaria infections could be acquired there, subsequently fuelling the malaria reservoir and infection risk for others in these areas, a concept that has been proposed previously [56]. Study clusters that include fishing beaches almost all appear to have higher risk because of outdoor occupations. For example the small cluster in the north and the smaller clusters west of the island, which fall within a malaria hot spot (Fig. 2b). In the northern part of the island there are also clusters with a reduced risk of malaria for outdoor occupation; these clusters lie in one of the malaria hot spots. The effect is not as large and is also less significant, but possibly an explanation here can be that in this area farming, also an outdoor occupation, is the dominant occupation, usually performed during the day when mosquitoes are less active. Nevertheless working outside at dawn and dusk becomes increasingly more important as a predictor of malaria risk as the mosquito vectors are recurrently reported to bite after sunrise and before sunset [33].

Socioeconomic status has often been linked with risk of malaria. Better schooling, improved housing and a higher income are commonly associated with reduced malaria risk [57]. On Rusinga, areas with a higher risk as well as areas with a lower risk for malaria when residing in the highest SES category are identified. The local patterns of SES show that a positive association with malaria mostly affects the central western part of the island and the tip in the north-east (orange clusters), with an increased risk of malaria. The south-eastern part (green clusters) of the island, by contrast, yield clusters that show a reduced risk of malaria among those with the highest SES.

Socioeconomic status itself does not affect malaria directly; hence the components of SES were further explored. It was found that in most of the clusters where high SES is associated with increased malaria risk, most farmland and dwellings are owned by the occupants while house structure is predominantly poor. This could suggest that variables as owning land and a house, indicators for being in a high SES class, do not necessarily directly relate to reduced malaria risk. Thus even though people are in the highest SES class, the house structure could allow for considerable malaria risk because there is poor protection against mosquitoes entering the house. A higher education level of the head of household could indicate that there is more financial freedom within the family. This can possibly result in a higher expenditure on health care and malaria prevention, which would presumably lead to reduced malaria risk. The components of location of kitchen and wall structure in this SES PCA are proxies of exposure to mosquitoes. When people cook outside during sunset and at night-time they may be exposed to outdoor-biting mosquitoes. Finally and interestingly SES did not have a strong (Fig. 5b) or significant relationship (Fig. 6b) with malaria in the hot spots (Fig. 2b). Thus, residing in a malaria hot spot was independent of house ownership, educational level or other SES factors.

A higher population density was associated with a slightly reduced risk of malaria in the GLR model, in keeping with previous findings from various studies in both urban and rural settings in Africa [58]. Higher population density has a large protective effect in some clusters farther from the lake and further from potential breeding sites, whereas the association between population density and malaria risk was positive in some clusters closer to the lake. It appears that the effect of a higher population density depended on proximity to possible breeding sites of malaria vectors near the lake shore. In a large simulation study [19] the dynamics of a spatially heterogeneous human and mosquito population was modelled and it was suggested that where there are few mosquitoes or breeding sites, the chance of receiving an infective bite is reduced in densely populated areas whereas the chance or receiving an infective bite is not reduced in sparsely populated areas. On the other hand, if there are many breeding sites and many mosquitoes close to a densely populated area, the chance of malaria transmission increases considerably compared to areas that are less densely populated where the chance or malaria transmission does not increase further with increasing mosquito numbers.

Other risk factors considered in the GLR model have all been suggested in previous literature as predictive for malaria risk. Remarkably, human age and mosquito counts as a proxy for exposure did not enter the final model. Young children (0–5 years) and adolescents typically have a higher risk of malaria because of different behaviour regarding malaria prevention and less well developed immune systems [59]. However, on Rusinga age was not significantly related to malaria, and there was no spatial heterogeneity in the effect of age on malaria. Furthermore, increased numbers of mosquitoes caught in some clusters were not accompanied by higher local prevalence. Screened eaves was not a significant predictor, but this can be explained by the fact that more than 90 % of the households did not have screened eaves and therefore there was insufficient information relating to the impact of this variable. There was a fairly homogenous coverage of bed nets and IRS activities across the island in the year prior to the present study. Bed nets continued to be used, but no further IRS treatments took place. This lack of variability could explain why number of bed nets and IRS coverage were not significantly associated with malaria. NDVI and TWI were also rather homogeneous over the island and therefore not important predictors for malaria. Finally, the average distance to a clinic did not play a role in this model. On this relatively small island, there are five health clinics or dispensaries, and even the households furthest away from a health clinic are at a walking distance of only 3 km.

An advantage of this study is firstly the assumption that non-stationarity of underlying risk factors for malaria can improve model fit considerably and can subsequently be used to explore geographically varying factors responsible for spatial patterns of malaria. Local outcomes and relationships can shed light on why malaria persists in certain areas. Secondly, as the data collected for this analysis serves as the baseline survey for a large vector control study, this analysis can assist in exploring further research and explain why the interventions may ultimately perform better in some areas than in others. One could consider increasing the intensity of available malaria interventions near fishing beaches at night, account for poor housing structures and reduce the number of traps in a densely populated area where high population density is associated with lower risk of malaria.

It is essential to understand the degree by which the results could be influenced by the unit of analysis. The use of discrete zones to perform spatial analysis is very common [60], but rather contradictory because geographical variation is a continuous process. Project clusters were defined and used to perform the intervention study, with the baseline malaria data described here. The number of clusters and population size per cluster were optimized and adopted for the rollout of the vector control intervention with optimal statistical power as well as community acceptance [61]. Creation of 81 clusters with an even number of households per cluster was calculated to provide sufficient generalizability and randomness to detect a possible difference in malaria incidence (T Smith, personal communication). As the intervention trial is analysed on basis of geographical divisions it was logical to use the same clusters for analysis of baseline data, which gave rise to this work. Spatial analyses are often performed on a similar scale at which this data was collected, for instance on village or county level [62]. Published work stresses that a societal or biological rationale is important when constructing discrete geographical zones. The rationale behind using the project clusters in this study is because it will be valuable to know what factors will have influenced the outcome of the vector intervention study which was conducted on this cluster scale. However, using different discrete clusters or cluster sizes or individual level data may yield slightly different outcomes. More detailed variation in coefficients is yielded when using smaller units and vice versa [47]. Additionally, when using an adaptive kernel function the radius of data included of local regression is variable. Also here it applies that smaller scale local regression usually leads to more variation in coefficients [63], and this mostly leads to weaker or stronger local relationships rather than reversed relationships. Nonetheless, when first performing a global linear regression, one can be confident that the risk factors obtained are important predictors of malaria and that subsequently the local coefficients of GWR are justified, despite of varying strengths of the relationships being influenced by the scale chosen [60].

Further limitations of this analysis are linked with the statistical methods used by GWR [64]. GWR has been criticized for lacking an integrated statistical framework because it represents a collective of local spatial regressions and a precise inference becomes imperfect. In understanding the varying coefficients one has to bear in mind that the coefficients that were estimated can be interpreted as an exploration and not as exact inference [62]. Since this issue was raised, significance tests have been developed to reduce uncertainty about the relationships identified using this approach. These local tests were incorporated in our analysis, showing areas where relationships were more significant than in other areas. Another concern raised regarding GWR is that the technique yields local effects that can be inflated because of residual spatial autocorrelation and multicollinearity. Residual spatial autocorrelation occurs when regression residuals cluster spatially, violating the assumption of independence in a linear regression model. Even though GWR accounts for this by adding a random error term for observations, coefficients can become inflated due to clustering of residuals. In this analysis much care was invested in examining and testing for RSA, minimizing possible uncertainty in coefficients resulting from RSA. Finally, in recent years another limitation of GWR was put forward; inflation of local coefficients because of local multicollinearity [65]. If predictor variables locally indicate the same patterns, their effect on the outcome variable can be overestimated. Since this problem was raised several tools have been developed to assess the extent of local multicollinearity [48]. In this analysis a measure of local multicollinearity by means of the condition number was incorporated, but it is concluded that this issue caused a negligible distorting effect on the local coefficients.

## Conclusion

In this study, geographically-varying risk factors for malaria were modelled. The spatial heterogeneity of malaria risk factors is explored rather than concluding upon perfect inferences. The study reveals that predictor variables for malaria vary geographically even over small distances of several kilometres. The exploration demonstrates that assuming stationarity of risk factors by means of a global statistical model ignores spatial components that can yield useful information and improve model fit. Being part of the highest SES, working outdoors (during night time) and population density were most predictive for malaria patterns on Rusinga Island. When considering SES as a risk factor for malaria one has to bear in mind that this depends on the local setting and the components included, hence results need to be interpreted with caution. All relationships with risk factors were spatially heterogeneous and these varying effects can be used to explore for what reasons vector intervention at the island possibly may have dissimilar effects in different areas.
